# Lumbar Ligament Sprain-degeneration and Prolapsed Lumbar Intervertebral Disc: A Frequent Missed Combination

**DOI:** 10.7759/cureus.3958

**Published:** 2019-01-25

**Authors:** Md Abu B Siddiq, Mohammad A Rahim, Md Tariqul I Khan, AHM Zakir H Shikder

**Affiliations:** 1 Physical Medicine and Rehabilitation, Brahmanbaria Medical College, Brahmanbaria, BGD; 2 Physical Medicine and Rehabilitation, Cox's Bazar Medical College, Chittagong, BGD; 3 Physical Medicine and Rehabilitation, Rajshahi Medical College, Rajshahi, BGD; 4 Dentistry, Bangabandhu Sheikh Mujib Medical University, Dhaka, BGD

**Keywords:** low back pain, lumbosacral region, intervertebral disc disease, intervertebral disc degeneration, ligament sprain

## Abstract

Low back pain (LBP) is a frequent complaint in the working milieu of pain physicians. Common LBP generators are the lumbar spine, soft tissues around the spine, and intra-abdominal viscera; however, in recent times, lumbar spine ligament (LL) degeneration is increasingly getting more coverage as an important LBP source. Among various LLs, interspinous and supraspinatus ligaments’ sprain-degeneration can perpetuate localized central LBP as described in the present case study. Localized LL sprain-degeneration in association with radiating LBP from prolapsed lumbar intervertebral disc (PLID) compressing adjacent nerve roots might further impair a patient's quality of life. In the present report, we describe both LL sprain-degeneration and PLID (a dual source of LBP) in a 26-year-old Bangladeshi woman; physicians often fail to notice this combination in their regular clinical practice.

## Introduction

Low back pain (LBP) is the second most common presentation of pain in general; hence in their day-to-day clinical practice pain physicians are accustomed to face various causes of it. Prevalence of LBP is rising and it can affect people from all walks of life at any point of their life time [1]. LBP can be classified either based on the structures involved [for example, facet arthropathy, prolapsed lumbar intervertebral disc (PLID) etc.] or based on the pattern of pathology (for example, inflammation, degeneration, infection, cancer). Among them, lumbar spine degeneration, especially in the elderly which could proliferate features unique for degenerative LBP is one of the most common; however, in recent times, accumulating evidence postulate that posterior lumbar spine ligaments (LLs) sprain-degeneration could also be a significant LBP contributor [2].

Among various LLs, pathology as of posterior LL, namely, lumbar interspinous and supraspinatus ligament sprain-degeneration are getting more focus in medical publications as one of the important LBP generators. In LL degeneration, patients usually present with central nonradiating LBP, aggravating during prolonged sitting and standing, forward bending, or even raising themselves from sitting posture while participating in activities of daily living (ADL) [2].Though exact pathophysiology is yet to unveil for the ailment, events impacting over the low back region including fall, heavy weight lifting, and poor posture might contribute to the disease process. In contrast, PLID compressing nerve roots vicinity produces radiating LBP which is one of the most important causes of LBP, contributes to patients’ disability and impaired quality of life as well.

When LBP is not specified, searching for yellow flag signs (Waddell's sign) for psychogenic LBP is important [3]. Sometimes LBP may be in association with red flag signs (intractable pain, fever, weight loss, history of radiotherapy, inflammatory LBP, vertebral body fracture, altered bowel-bladder habit, neuro-deficit, saddle anesthesia, etc.) of some red flag conditions, such as malignancy, pyogenic (*Staphylococcus aureus*), chronic bone infection (mycobacterium tuberculosis), and inflammatory arthritis that deserves special attention in terms of their correct diagnosis and treatment [3].

Both LL sprain and PLID could respond to commonly used pain medicines, namely, analgesic (opioid/nonopioids) and nonsteroidal anti-inflammatory drugs (NSAIDs). Besides physical therapy, therapeutic spinal exercises could also be effective. However, intra-lesional (IL) steroid-lidocaine combination and epidural steroid could provide even better pain relief in LL sprain and PLID, respectively [2, 4].

The LL degeneration is a rare LBP association and in combination with PLID, the condition is yet to be surfaced on medical literature. In the present case study, we take the privilege of describing both conditions in the same patient, in order to make conscious both the patients and scientific community about the possibility of this LBP association. And we did it with patient’s prior informed consent.

## Case presentation

A 26-year-old, overweight (BMI—27.9 kg/m^2^), Asian-Bangladeshi female, presented with the complaint of severe LBP that preferably localized over the lower lumbar spine segment, aggravated with prolonged sitting, bending forward from her waist, even after the usual domestic chores for years. There were increased pain bouts in the last few weeks with a patient’s reported global pain assessment score 9/10, based on VAS (visual analog scale,10 cm) scale. The pain was well localized to the spine and not associated with significant morning stiffness as seen in inflammatory LBP. History of recent trauma, nocturnal fever, cough, weight loss, urinary incontinence, or altered bowel habit was also insignificant. The patient claimed, five years ago, multiple lumbar spine pricks that were performed during spinal anesthesia at her first cesarean section could perpetuate the pathology; however, she failed to register doctors’ attention regarding the problem. Considering all these facts, the initial clinical diagnosis ‘lumbar ligament sprain’ (LL sprain) was made, and we recommended a magnetic resonance imaging (MRI) of the lumbosacral spine for further analysis. At that time, the patient was managed conservatively including local application of ice, etoricoxib, and proton pump inhibitor (esomeprazole). Unfortunately, until her second visit to us, we lost the patient to follow up for some months. 

**Figure 1:**
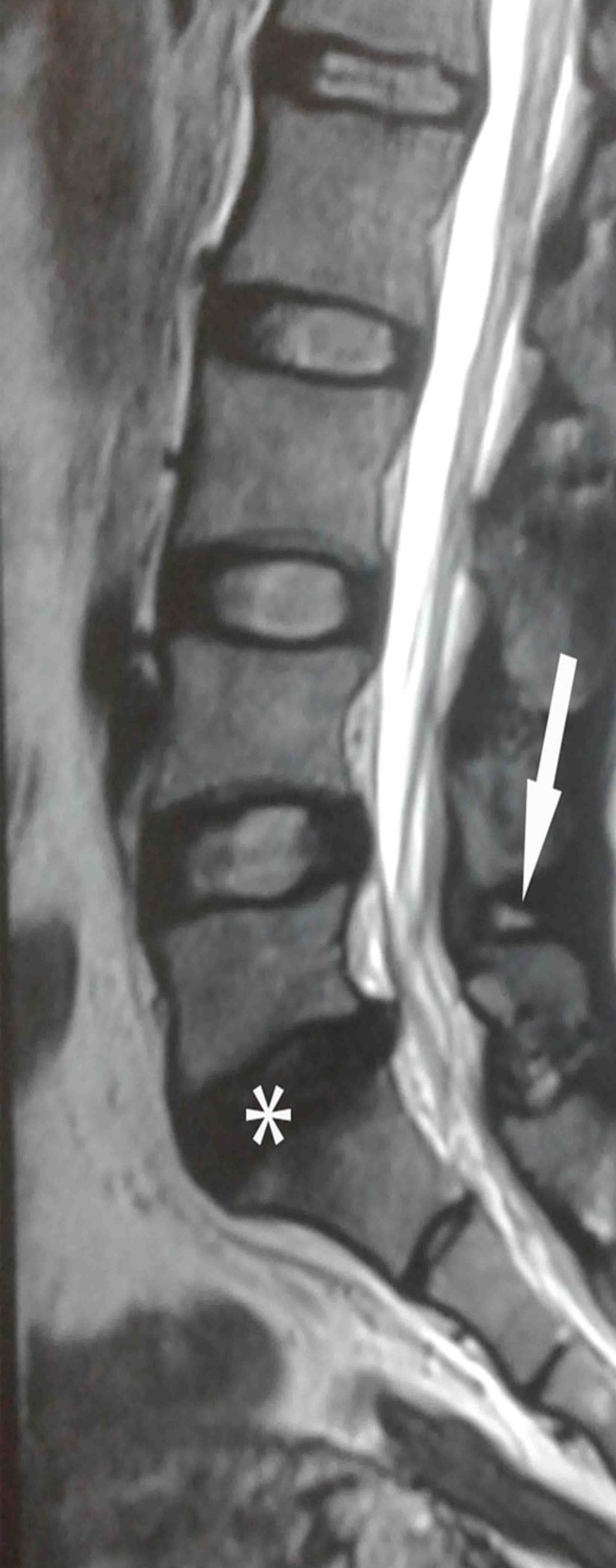
Magnetic resonance imaging (MRI) of lumbosacral spine. On longitudinal mid-sagittal scanning of the lumbosacral spine, T2-image depicts intervertebral disc degeneration and herniation between L5 and S1 vertebral bodies (asterisk) and inter-spinous ligament degeneration (high intensity on T2 image) (white arrow).

However, a few months later, all of a sudden, she developed severe radiating LBP with crunching, following lifting some weights on her back, followed by limited movements, impairing ADLs, though without alteration of usual bowel-bladder habits. PLID at L5-S1 level with corresponding nerve roots compression had been diagnosed based on both clinical and MRI findings (Figure 1). As the pain was intractable, she reported to the emergency orthopedic department and was managed by a consultant with a single shot of unguided lumbar inter-laminar epidural steroid injection that eventually relieved her radiating LBP significantly (more than 50% pain had relieved) within the next five days. 

**Figure 2:**
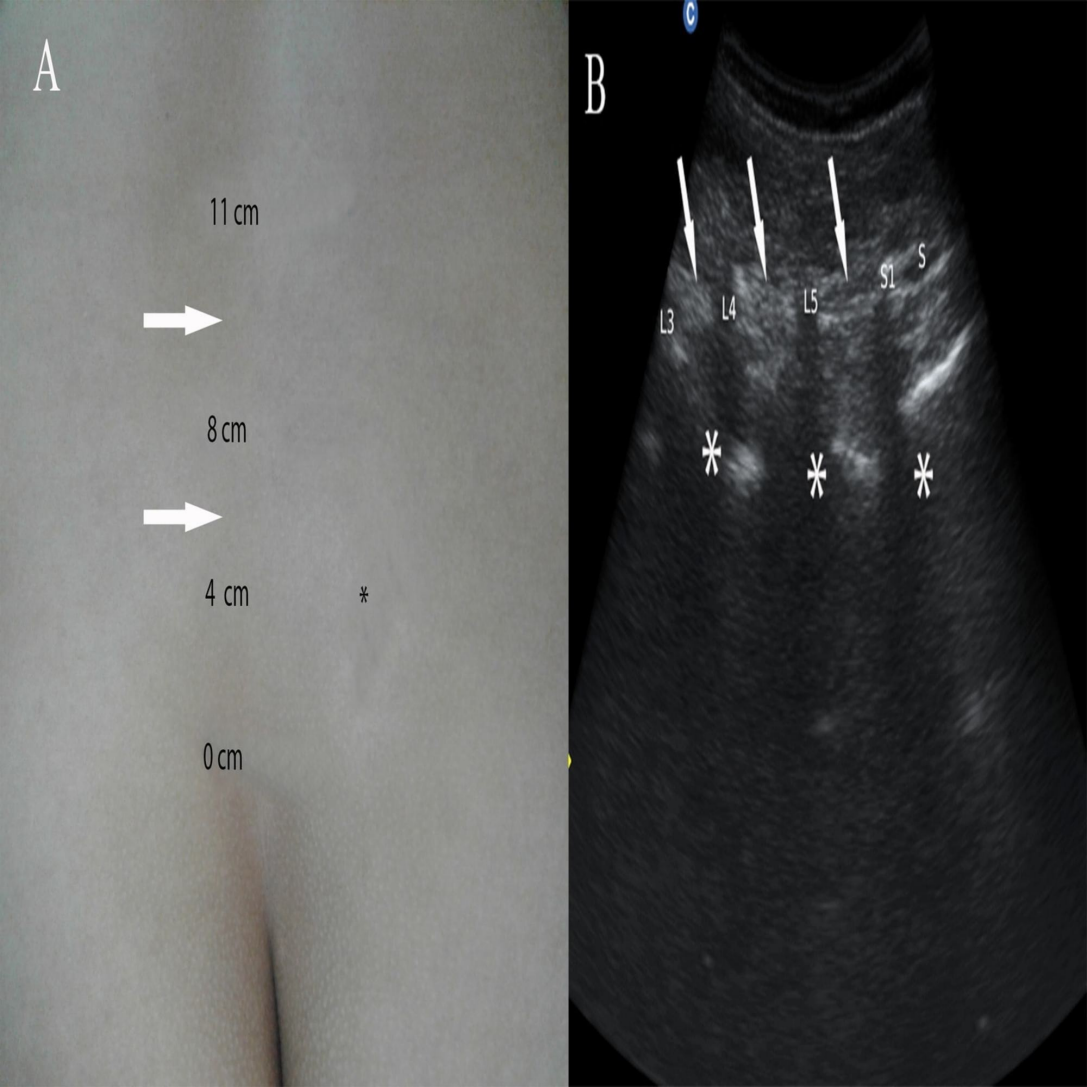
Surface marking demonstrates distance of different lumbar spine bony landmarks from sacral cornua measured under high-frequency (curvilinear) ultrasonogram. A. Surface marking demonstrates distance of different lumbar spine landmarks revealing under high-frequency (curvilinear) ultrasonogram, where 0, 4, 8, 11 cm, respectively, indicates level of sacral cornua, sacral promontory, L5 spinous process, and L4 spinous process; asterisk indicates sacroiliac joint (right). B. On ultrasonogram, asterisks reveal posterior acoustic shadowing of sacral 1, L5, L4, L3 spinous process (from right to left), and white arrow indicates hyperechoic interspinous spaces between hypoechoic posterior acoustic shadowing for spinous processes.

Later on, in her third visit to our clinic, we found the patient with localized lumbar spine pain as was seen in her first visit, though with a reduced VAS for pain (7/10 cm) score. On physical examination, no significant abnormalities were revealed, except a focal, well-localized, tender area in between L3 and L4 spinous processes, measuring 11 cm cranial to sacral cornua, under high-frequency musculoskeletal ultrasonogram (curvilinear probe, 5 MHz) (Chison CEO1, Guangdong, PR China) (Figures 2A-2B). Alongside, MRI evidences of lumbar disc degeneration-herniation at the L5/S1 level, mid-sagittal MRI-T2-weighted image revealed high-signal intensity at L5/S1 interspinous ligament representing degeneration, with marked narrowing of the interspinous ligament at L3/4, L4/5/S1 levels (Figure 1). Second time, LL-degeneration diagnosis with MRI-evidenced PLID (clinically lenient) was made. Finally, injection lidocaine (2%) was placed at the maximum tender area over the spine that provided more than 50% immediate pain relief. The patient was recommended to do regular spine muscle strengthening exercise. 

## Discussion

There are five common lumbar ligaments of clinical significance. Interspinous and supraspinatous ligaments are two of the important lumbar ligaments [5]. Interspinous ligament gradually becomes thicker from cranial to caudal spine segments and blends anteriorly with ligamentum flavum and posteriorly with supraspinatous ligament and together they limit excess lumbar spine forward bending. As the day progresses, both posterior spinal ligaments undergo degeneration primarily because of aging and secondary to the previous injury, prolonged static spine posture, surgery, weight lifting, and abnormal body habitus. In the present study subject, though we did not find the exact reason for LL-degeneration, previous multiple lumbar pricks during spinal anesthesia might have induced the pathology. Besides, increased body weight and repeated forward bending might have impaired sprained ligament to get repaired, with resultant painful fatty degeneration or intense cellular proliferation with vascular invasion of the injured ligament contributing localized spine pain. However, literature supporting spinal anesthesia-mediated LL sprain/degeneration is lacking. In a previously published report, Goobar et al. demonstrated clinical-pathological-MRI evidence of lumbar ligament sprain-degeneration [5]. In another clinical report, Kulkarni and Ramyashree mentioned about the LL sprain's possibility following spinal anesthesia in a young male who had undergone circumcision, nevertheless they did not classify the LL sprain changes under MRI or histopathology [2]. So, more research with a large number of study participants is required to measure whether any association exists between LL sprain-degeneration and previous spine pricks.

In their study, Keorochana and colleagues demonstrated different grades of interspinous ligament degeneration (based on MRI findings) as reported below [6]—A, low or iso-signal intensity on T1- and T2-weighted images or mixed signal intensity; B, high-signal intensity on T1-weighted images and high-signal intensity on T2-weighted images; C, low-signal intensity on T1-weighted images and high-signal intensity on T2-weighted images; D, low- or iso-signal intensity on T1- and T2-weighted images with hypertrophy or marrow alteration within spinous processes or narrowing of interspinous ligament interval. Here, in the present case, we see, high-signal intensity on T2-weighted images (we did not have a T1-weighted image), so the studied patient may fall to either grade-B or grade-C category. On histopathology, the following changes can reveal a degenerated ligament—fatty degeneration, profuse cellular infiltration with vascular invasion (inflammatory interspinous bursitis mimicking Basstrup’s disease), or massive fibrosis, with overlapping histopathology features or progressive loss of interspinous space in some instances [6]. Fatty degeneration represents MRI stage-B, whereas vascular/inflammatory changes represent MRI stage-C. However, like Keorochana et al., we could not perform a histopathological analysis of the ligamentous lesion and accept it as our study limitation. The MRI findings here in the present study represent the LL sprain/degeneration cascades as described in previous radio-pathological investigations [5-6].

Prolapsed lumbar intervertebral disc is frequent with heavy weight lifting, especially in elderly people with prior disc degeneration. In the present study, PLID with vicinity nerve roots compression has been reported in association with LL sprain-degeneration simultaneously in a young woman [7]. In chronic LBP, variable levels of evidence favor use of analgesics (opioids), NSAIDs, pregabalin, duloxetine, amitriptyline, therapeutic exercises (McKenzie back extension exercise, spinal stabilization exercise, and spinal muscle strengthening exercise), ergonomic training, acupuncture, mindfulness-based stress reduction, tai chi, yoga, progressive relaxation, electromyography biofeedback, transcutaneous electrical nerve stimulation, low-level laser therapy, cognitive behavioral therapy, spinal manipulation, and ADL modifications (avoidance of prolonged static posture, restriction of too much forward bending, avoidance of excess weight lifting) in mitigating patients’ symptoms [7-8]. However, we are yet to have appropriate diagnostic and therapeutic approaches for LL sprain—conventional pain killers relieve pain up to a certain level, whereas IL steroid-lidocaine injection combination could provide even better pain relief as is seen in the current study subject [2, 9]. As platelet rich plasma (PRP) is found useful in various regional soft tissue rheumatisms, same result could be echoed in prospective, longitudinal research with PRP in LL sprain-degeneration and is warranted [10]. IL steroid injection sometimes could be complicated with needle over penetration and localized superficial and deep infection; by fortune such events had not been registered in our present study subject.

In this study, we unveil both LL sprain-degeneration and PLID in the same patient, first time ever in medical literature. Still we are not certain about few issues regarding LL sprain-degeneration—why PLID has developed on a previously sprained LL or whether any relation prevails between LL sprain and future PLID or whether there is any link between the previous repeated lumbar pricks and LL sprain manifestation. It seems, here in the present study subject, painful LL sprain causes alteration of biomechanics of lumbosacral spine segment with resultant degeneration of adjacent intervertebral disc that later gets prolapsed even on minimum impact over the low back [11]. We are yet to know exactly what would be the approximate duration between LL degeneration and anticipated disc herniation. Hopefully, researchers in the field will address these issues through their future endeavors.

## Conclusions

Lumbar ligament degeneration and herniated lumbar intervertebral disc cause localized and radiating LBP, respectively. However, among them, the previous one is rarely reported and in association with PLID, LL sprain-degeneration is even rarer. Besides, lack of physicians’ concern about LL sprain-degeneration causes diagnostic delay and induces launching treatment that is inappropriate for it as well. We recommend further study with a large number of patients with LL sprain addressing the following areas—what is the relation between prior LL sprain-degeneration and future PLID; how many patients have both lumbar ligament sprain and PLID simultaneously; how prevalent is LL-sprain among chronic LBP sufferers; what could be the approximate duration between lumbar ligament sprain and anticipated PLID; or whether occupation gives rise to lumbar ligament sprain.
